# Supplementary material for: Atomistic simulation of protein evolution reveals sequence covariation and time-dependent fluctuations of site-specific substitution rates
Source: PLoS Comput Biol. 2023 Mar 24;19(3):e1010262. doi: 10.1371/journal.pcbi.1010262 (PMC10075473; doi:10.1371/journal.pcbi.1010262)
Supplement: S1 Text — (PDF) [file pcbi.1010262.s001.pdf]

S1_text:

"**Atomistic simulation of protein evolution reveals sequence covariation and time-dependent fluctuations of site-specific substitution rates**" - Command lines and run examples

RosettaEvolve is a set of methods used to simulated the evolution of proteins using an atomistic energy function and a stability-based fitness function. RosettaEvolve uses functionality of Rosetta for simulation of protein evolution and consists of a set of Rosetta movers, primarily NucleotideMutation mover and EvolutionaryDynamics mover. In this study we have used the xml scripting language of Rosetta to run simulations, although pyrosetta could also be employed.

In this study four RosettaEvolve runs were carried out: 1) An equilibrium run to create the initial structures and sequences used in the simulation. 2) Trajectories starting from the equilibrated sequences and structures along a single branch 3) Trajectories involving the introduction of a single non-synonymous mutations, repeated according to the number of sites in the protein. 4) A tree crawling simulation to generate a phylogenetic tree.

Details and example data are found in the repository associated with this study, https://github.com/Andre-lab/RosettaEvolve. Below some of the example scripts are shown, in addition to information how these simulations relate to the results in the manuscript.

1) Equilibrium run to create the initial structure and sequence

Starting from an energy refined atomic structure equilibration simulations are carried out with a given energy offset to generate starting structures and sequences for production runs. These simulations were run with the following parameters:

kappa=2.7
rho=0.1
The energy function used was "beta_nov16_cart".

The data for the equilibrium simulation is described in the section "*Equilibration of trajectories*" in the manuscript and presented in **Figure 2** in the main text.

RosettaEvolve Equilibration run example:

rosetta_scripts.default.linuxgccrelease -s in/5azu.pdb -parser:protocol equilibrate_sequence.xml @flags -overwrite -out:prefix pdbs/5azu. -parser:script_vars id=5azu e_delta=-472.566 offset=-472.566 n_trials=127

with the following XML script, equilibrate_sequence.xml:

```
<ROSETTASCRIPTS>
    <SCOREFXNS>
        <ScoreFunction name="beta_nov16_cart" weights="beta_nov16_cart"/>
    </SCOREFXNS>

  <FILTERS>
        <ScoreType name="total_score" scorefxn="beta_nov16_cart" score_type="total_score" confidence="0" threshold="100000"/>
        <Sigmoid name="stability" filter="total_score" steepness="0.86" offset="%%offset%%" negate="0"/>
```

```
</FILTERS>

<MOVERS>
<NucleotideMutation name="mut" allow_silent="0" bbnbrs="0" fast_relax_cycles="1" flexbb="0" dualspace="1" kappa="2.7" rho="0.1"
scorefxn="beta_nov16_cart"/>
<NucleotideMutation name="measure_energies0" bbnbrs="0" score_file="ranks/bl_0_%%e_delta%%_%%id%%.txt" allow_silent="1"
fast_relax_cycles="1" measure_e_dist_at_site="9999" flexbb="0" dualspace="1" scorefxn="beta_nov16_cart"/>
<NucleotideMutation name="measure_energies1" bbnbrs="0" score_file="ranks/bl_1_%%e_delta%%_%%id%%.txt" allow_silent="1"
fast_relax_cycles="1" measure_e_dist_at_site="9999" flexbb="0" dualspace="1" scorefxn="beta_nov16_cart"/>
<NucleotideMutation name="measure_energies2" bbnbrs="0" score_file="ranks/bl_2_%%e_delta%%_%%id%%.txt" allow_silent="1"
fast_relax_cycles="1" measure_e_dist_at_site="9999" flexbb="0" dualspace="1" scorefxn="beta_nov16_cart"/>
<NucleotideMutation name="measure_energies3" bbnbrs="0" score_file="ranks/bl_3_%%e_delta%%_%%id%%.txt" allow_silent="1"
fast_relax_cycles="1" measure_e_dist_at_site="9999" flexbb="0" dualspace="1" scorefxn="beta_nov16_cart"/>
<NucleotideMutation name="measure_energies4" bbnbrs="0" score_file="ranks/bl_4_%%e_delta%%_%%id%%.txt" allow_silent="1"
fast_relax_cycles="1" measure_e_dist_at_site="9999" flexbb="0" dualspace="1" scorefxn="beta_nov16_cart"/>
<NucleotideMutation name="measure_energies5" bbnbrs="0" score_file="ranks/bl_5_%%e_delta%%_%%id%%.txt" allow_silent="1"
fast_relax_cycles="1" measure_e_dist_at_site="9999" flexbb="0" dualspace="1" scorefxn="beta_nov16_cart"/>
<NucleotideMutation name="measure_energies6" bbnbrs="0" score_file="ranks/bl_6_%%e_delta%%_%%id%%.txt" allow_silent="1"
fast_relax_cycles="1" measure_e_dist_at_site="9999" flexbb="0" dualspace="1" scorefxn="beta_nov16_cart"/>
<NucleotideMutation name="measure_energies7" bbnbrs="0" score_file="ranks/bl_7_%%e_delta%%_%%id%%.txt" allow_silent="1"
fast_relax_cycles="1" measure_e_dist_at_site="9999" flexbb="0" dualspace="1" scorefxn="beta_nov16_cart"/>
<NucleotideMutation name="measure_energies8" bbnbrs="0" score_file="ranks/bl_8_%%e_delta%%_%%id%%.txt" allow_silent="1"
fast_relax_cycles="1" measure_e_dist_at_site="9999" flexbb="0" dualspace="1" scorefxn="beta_nov16_cart"/>
<NucleotideMutation name="measure_energies9" bbnbrs="0" score_file="ranks/bl_9_%%e_delta%%_%%id%%.txt" allow_silent="1"
fast_relax_cycles="1" measure_e_dist_at_site="9999" flexbb="0" dualspace="1" scorefxn="beta_nov16_cart"/>
<NucleotideMutation name="measure_energies10" bbnbrs="0" score_file="ranks/bl_10_%%e_delta%%_%%id%%.txt" allow_silent="1"
fast_relax_cycles="1" measure_e_dist_at_site="9999" flexbb="0" dualspace="1" scorefxn="beta_nov16_cart"/>
NucleotideMutation name="measure_energies_0" bbnbrs="0" score_file="trajs/energies_trial_0_%%id%%.txt" allow_silent="1" fast_relax_cycles="1"
measure_e_dist_at_site="9999" flexbb="0" dualspace="1" scorefxn="beta_nov16_cart"/>
GenericMonteCarlo name="pre_minimization" reset_baselines="0" mover_name="mut" keep_filters="1" recover_low="1" preapply="0" drift="1"
progress_file="trajs/preminimzation.%%id%%.txt" filter_name="total_score" trials="12000"/>
<DumpPdb name="dump_1"  fname="dumps/%%id%%_%%e_delta%%_bl_1.pdb" scorefxn="beta_nov16_cart"/>
<DumpPdb name="dump_2"  fname="dumps/%%id%%_%%e_delta%%_bl_2.pdb" scorefxn="beta_nov16_cart"/>
<DumpPdb name="dump_3"  fname="dumps/%%id%%_%%e_delta%%_bl_3.pdb" scorefxn="beta_nov16_cart"/>
<DumpPdb name="dump_4"  fname="dumps/%%id%%_%%e_delta%%_bl_4.pdb" scorefxn="beta_nov16_cart"/>
<DumpPdb name="dump_5"  fname="dumps/%%id%%_%%e_delta%%_bl_5.pdb" scorefxn="beta_nov16_cart"/>
<DumpPdb name="dump_6"  fname="dumps/%%id%%_%%e_delta%%_bl_6.pdb" scorefxn="beta_nov16_cart"/>
<DumpPdb name="dump_7"  fname="dumps/%%id%%_%%e_delta%%_bl_7.pdb" scorefxn="beta_nov16_cart"/>
<DumpPdb name="dump_8"  fname="dumps/%%id%%_%%e_delta%%_bl_8.pdb" scorefxn="beta_nov16_cart"/>
<DumpPdb name="dump_9"  fname="dumps/%%id%%_%%e_delta%%_bl_9.pdb" scorefxn="beta_nov16_cart"/>
<DumpPdb name="dump_10" fname="dumps/%%id%%_%%e_delta%%_bl_10.pdb" scorefxn="beta_nov16_cart"/>

<EvolutionaryDynamics name="evolve_0"  ignore_fitness_equilibrium_check="1" reset_baselines="0" max_accepted_trials="%%n_trials%%"
mover_name="mut" recover_low="0" preapply="0" drift="1" progress_file="trajs/progress_%%id%%_%%e_delta%%_bl_0.txt" filter_name="stability"
trials="1000000" scorefxn_name="beta_nov16_cart" steepness="0.86" population_size="15848" offset="%%offset%%"/>
<EvolutionaryDynamics name="evolve_1"  ignore_fitness_equilibrium_check="1" reset_baselines="0" max_accepted_trials="%%n_trials%%"
mover_name="mut" recover_low="0" preapply="0" drift="1" progress_file="trajs/progress_%%id%%_%%e_delta%%_bl_1.txt" filter_name="stability"
trials="1000000" scorefxn_name="beta_nov16_cart" steepness="0.86" population_size="15848" offset="%%offset%%"/>
<EvolutionaryDynamics name="evolve_2"  ignore_fitness_equilibrium_check="1" reset_baselines="0" max_accepted_trials="%%n_trials%%"
mover_name="mut" recover_low="0" preapply="0" drift="1" progress_file="trajs/progress_%%id%%_%%e_delta%%_bl_2.txt" filter_name="stability"
trials="1000000" scorefxn_name="beta_nov16_cart" steepness="0.86" population_size="15848" offset="%%offset%%"/>
<EvolutionaryDynamics name="evolve_3"  ignore_fitness_equilibrium_check="1" reset_baselines="0" max_accepted_trials="%%n_trials%%"
mover_name="mut" recover_low="0" preapply="0" drift="1" progress_file="trajs/progress_%%id%%_%%e_delta%%_bl_3.txt" filter_name="stability"
trials="1000000" scorefxn_name="beta_nov16_cart" steepness="0.86" population_size="15848" offset="%%offset%%"/>
<EvolutionaryDynamics name="evolve_4"  ignore_fitness_equilibrium_check="1" reset_baselines="0" max_accepted_trials="%%n_trials%%"
mover_name="mut" recover_low="0" preapply="0" drift="1" progress_file="trajs/progress_%%id%%_%%e_delta%%_bl_4.txt" filter_name="stability"
trials="1000000" scorefxn_name="beta_nov16_cart" steepness="0.86" population_size="15848" offset="%%offset%%"/>
<EvolutionaryDynamics name="evolve_5"  ignore_fitness_equilibrium_check="1" reset_baselines="0" max_accepted_trials="%%n_trials%%"
mover_name="mut" recover_low="0" preapply="0" drift="1" progress_file="trajs/progress_%%id%%_%%e_delta%%_bl_5.txt" filter_name="stability"
trials="1000000" scorefxn_name="beta_nov16_cart" steepness="0.86" population_size="15848" offset="%%offset%%"/>
<EvolutionaryDynamics name="evolve_6"  ignore_fitness_equilibrium_check="1" reset_baselines="0" max_accepted_trials="%%n_trials%%"
mover_name="mut" recover_low="0" preapply="0" drift="1" progress_file="trajs/progress_%%id%%_%%e_delta%%_bl_6.txt" filter_name="stability"
trials="1000000" scorefxn_name="beta_nov16_cart" steepness="0.86" population_size="15848" offset="%%offset%%"/>
<EvolutionaryDynamics name="evolve_7"  ignore_fitness_equilibrium_check="1" reset_baselines="0" max_accepted_trials="%%n_trials%%"
mover_name="mut" recover_low="0" preapply="0" drift="1" progress_file="trajs/progress_%%id%%_%%e_delta%%_bl_7.txt" filter_name="stability"
trials="1000000" scorefxn_name="beta_nov16_cart" steepness="0.86" population_size="15848" offset="%%offset%%"/>
<EvolutionaryDynamics name="evolve_8"  ignore_fitness_equilibrium_check="1" reset_baselines="0" max_accepted_trials="%%n_trials%%"
mover_name="mut" recover_low="0" preapply="0" drift="1" progress_file="trajs/progress_%%id%%_%%e_delta%%_bl_8.txt" filter_name="stability"
trials="1000000" scorefxn_name="beta_nov16_cart" steepness="0.86" population_size="15848" offset="%%offset%%"/>
<EvolutionaryDynamics name="evolve_9"  ignore_fitness_equilibrium_check="1" reset_baselines="0" max_accepted_trials="%%n_trials%%"
mover_name="mut" recover_low="0" preapply="0" drift="1" progress_file="trajs/progress_%%id%%_%%e_delta%%_bl_9.txt" filter_name="stability"
trials="1000000" scorefxn_name="beta_nov16_cart" steepness="0.86" population_size="15848" offset="%%offset%%"/>
```

```
  <EvolutionaryDynamics name="evolve_10" ignore_fitness_equilibrium_check="1" reset_baselines="0" max_accepted_trials="%%n_trials%%"
mover_name="mut" recover_low="0" preapply="0" drift="1" progress_file="trajs/progress_%%id%%_%%e_delta%%_bl_10.txt" filter_name="stability"
trials="1000000" scorefxn_name="beta_nov16_cart" steepness="0.86" population_size="15848" offset="%%offset%%"/>

 </MOVERS>
                                                                                                    23,5        Top
<ROSETTASCRIPTS>
     <SCOREFXNS>
          <ScoreFunction name="beta_nov16_cart" weights="beta_nov16_cart"/>
     </SCOREFXNS>

 <FILTERS>
          <ScoreType name="total_score" scorefxn="beta_nov16_cart" score_type="total_score" confidence="0" threshold="100000"/>
          <Sigmoid name="stability" filter="total_score" steepness="0.86" offset="%%offset%%" negate="0"/>
     </FILTERS>

 <MOVERS>
  <NucleotideMutation name="mut" allow_silent="0" bbnbrs="0" fast_relax_cycles="1" flexbb="0" dualspace="1" kappa="2.7" rho="0.1"
scorefxn="beta_nov16_cart"/>
   <NucleotideMutation name="measure_energies0" bbnbrs="0" score_file="ranks/bl_0_%%e_delta%%_%%id%%.txt" allow_silent="1"
fast_relax_cycles="1" measure_e_dist_at_site="9999" flexbb="0" dualspace="1" scorefxn="beta_nov16_cart"/>
  <NucleotideMutation name="measure_energies1" bbnbrs="0" score_file="ranks/bl_1_%%e_delta%%_%%id%%.txt" allow_silent="1"
fast_relax_cycles="1" measure_e_dist_at_site="9999" flexbb="0" dualspace="1" scorefxn="beta_nov16_cart"/>
  <NucleotideMutation name="measure_energies2" bbnbrs="0" score_file="ranks/bl_2_%%e_delta%%_%%id%%.txt" allow_silent="1"
fast_relax_cycles="1" measure_e_dist_at_site="9999" flexbb="0" dualspace="1" scorefxn="beta_nov16_cart"/>
  <NucleotideMutation name="measure_energies3" bbnbrs="0" score_file="ranks/bl_3_%%e_delta%%_%%id%%.txt" allow_silent="1"
fast_relax_cycles="1" measure_e_dist_at_site="9999" flexbb="0" dualspace="1" scorefxn="beta_nov16_cart"/>
  <NucleotideMutation name="measure_energies4" bbnbrs="0" score_file="ranks/bl_4_%%e_delta%%_%%id%%.txt" allow_silent="1"
fast_relax_cycles="1" measure_e_dist_at_site="9999" flexbb="0" dualspace="1" scorefxn="beta_nov16_cart"/>
  <NucleotideMutation name="measure_energies5" bbnbrs="0" score_file="ranks/bl_5_%%e_delta%%_%%id%%.txt" allow_silent="1"
fast_relax_cycles="1" measure_e_dist_at_site="9999" flexbb="0" dualspace="1" scorefxn="beta_nov16_cart"/>
  <NucleotideMutation name="measure_energies6" bbnbrs="0" score_file="ranks/bl_6_%%e_delta%%_%%id%%.txt" allow_silent="1"
fast_relax_cycles="1" measure_e_dist_at_site="9999" flexbb="0" dualspace="1" scorefxn="beta_nov16_cart"/>
  <NucleotideMutation name="measure_energies7" bbnbrs="0" score_file="ranks/bl_7_%%e_delta%%_%%id%%.txt" allow_silent="1"
fast_relax_cycles="1" measure_e_dist_at_site="9999" flexbb="0" dualspace="1" scorefxn="beta_nov16_cart"/>
  <NucleotideMutation name="measure_energies8" bbnbrs="0" score_file="ranks/bl_8_%%e_delta%%_%%id%%.txt" allow_silent="1"
fast_relax_cycles="1" measure_e_dist_at_site="9999" flexbb="0" dualspace="1" scorefxn="beta_nov16_cart"/>
  <NucleotideMutation name="measure_energies9" bbnbrs="0" score_file="ranks/bl_9_%%e_delta%%_%%id%%.txt" allow_silent="1"
fast_relax_cycles="1" measure_e_dist_at_site="9999" flexbb="0" dualspace="1" scorefxn="beta_nov16_cart"/>
  <NucleotideMutation name="measure_energies10" bbnbrs="0" score_file="ranks/bl_10_%%e_delta%%_%%id%%.txt" allow_silent="1"
fast_relax_cycles="1" measure_e_dist_at_site="9999" flexbb="0" dualspace="1" scorefxn="beta_nov16_cart"/>
  NucleotideMutation name="measure_energies_0" bbnbrs="0" score_file="trajs/energies_trial_0_%%id%%.txt" allow_silent="1" fast_relax_cycles="1"
measure_e_dist_at_site="9999" flexbb="0" dualspace="1" scorefxn="beta_nov16_cart"/>
  GenericMonteCarlo name="pre_minimization" reset_baselines="0" mover_name="mut" keep_filters="1" recover_low="1" preapply="0" drift="1"
progress_file="trajs/preminimzation.%%id%%.txt" filter_name="total_score" trials="12000"/>
     <DumpPdb name="dump_1"  fname="dumps/%%id%%_%%e_delta%%_bl_1.pdb" scorefxn="beta_nov16_cart"/>
     <DumpPdb name="dump_2"  fname="dumps/%%id%%_%%e_delta%%_bl_2.pdb" scorefxn="beta_nov16_cart"/>
     <DumpPdb name="dump_3"  fname="dumps/%%id%%_%%e_delta%%_bl_3.pdb" scorefxn="beta_nov16_cart"/>
     <DumpPdb name="dump_4"  fname="dumps/%%id%%_%%e_delta%%_bl_4.pdb" scorefxn="beta_nov16_cart"/>
     <DumpPdb name="dump_5"  fname="dumps/%%id%%_%%e_delta%%_bl_5.pdb" scorefxn="beta_nov16_cart"/>
     <DumpPdb name="dump_6"  fname="dumps/%%id%%_%%e_delta%%_bl_6.pdb" scorefxn="beta_nov16_cart"/>
     <DumpPdb name="dump_7"  fname="dumps/%%id%%_%%e_delta%%_bl_7.pdb" scorefxn="beta_nov16_cart"/>
     <DumpPdb name="dump_8"  fname="dumps/%%id%%_%%e_delta%%_bl_8.pdb" scorefxn="beta_nov16_cart"/>
     <DumpPdb name="dump_9"  fname="dumps/%%id%%_%%e_delta%%_bl_9.pdb" scorefxn="beta_nov16_cart"/>
     <DumpPdb name="dump_10" fname="dumps/%%id%%_%%e_delta%%_bl_10.pdb" scorefxn="beta_nov16_cart"/>

  <EvolutionaryDynamics name="evolve_0"  ignore_fitness_equilibrium_check="1" reset_baselines="0" max_accepted_trials="%%n_trials%%"
mover_name="mut" recover_low="0" preapply="0" drift="1" progress_file="trajs/progress_%%id%%_%%e_delta%%_bl_0.txt" filter_name="stability"
trials="100000
00" scorefxn_name="beta_nov16_cart" steepness="0.86" population_size="15848" offset="%%offset%%"/>
  <EvolutionaryDynamics name="evolve_1"  ignore_fitness_equilibrium_check="1" reset_baselines="0" max_accepted_trials="%%n_trials%%"
mover_name="mut" recover_low="0" preapply="0" drift="1" progress_file="trajs/progress_%%id%%_%%e_delta%%_bl_1.txt" filter_name="stability"
trials="100000
00" scorefxn_name="beta_nov16_cart" steepness="0.86" population_size="15848" offset="%%offset%%"/>
  <EvolutionaryDynamics name="evolve_2"  ignore_fitness_equilibrium_check="1" reset_baselines="0" max_accepted_trials="%%n_trials%%"
mover_name="mut" recover_low="0" preapply="0" drift="1" progress_file="trajs/progress_%%id%%_%%e_delta%%_bl_2.txt" filter_name="stability"
trials="100000
00" scorefxn_name="beta_nov16_cart" steepness="0.86" population_size="15848" offset="%%offset%%"/>
  <EvolutionaryDynamics name="evolve_3"  ignore_fitness_equilibrium_check="1" reset_baselines="0" max_accepted_trials="%%n_trials%%"
mover_name="mut" recover_low="0" preapply="0" drift="1" progress_file="trajs/progress_%%id%%_%%e_delta%%_bl_3.txt" filter_name="stability"
trials="100000
00" scorefxn_name="beta_nov16_cart" steepness="0.86" population_size="15848" offset="%%offset%%"/>
```

```
  <EvolutionaryDynamics name="evolve_4"  ignore_fitness_equilibrium_check="1" reset_baselines="0" max_accepted_trials="%%n_trials%%"
mover_name="mut" recover_low="0" preapply="0" drift="1" progress_file="trajs/progress_%%id%%_%%e_delta%%_bl_4.txt" filter_name="stability"
trials="100000
00" scorefxn_name="beta_nov16_cart" steepness="0.86" population_size="15848" offset="%%offset%%"/>
  <EvolutionaryDynamics name="evolve_5"  ignore_fitness_equilibrium_check="1" reset_baselines="0" max_accepted_trials="%%n_trials%%"
mover_name="mut" recover_low="0" preapply="0" drift="1" progress_file="trajs/progress_%%id%%_%%e_delta%%_bl_5.txt" filter_name="stability"
trials="100000
00" scorefxn_name="beta_nov16_cart" steepness="0.86" population_size="15848" offset="%%offset%%"/>
  <EvolutionaryDynamics name="evolve_6"  ignore_fitness_equilibrium_check="1" reset_baselines="0" max_accepted_trials="%%n_trials%%"
mover_name="mut" recover_low="0" preapply="0" drift="1" progress_file="trajs/progress_%%id%%_%%e_delta%%_bl_6.txt" filter_name="stability"
trials="100000
00" scorefxn_name="beta_nov16_cart" steepness="0.86" population_size="15848" offset="%%offset%%"/>
  <EvolutionaryDynamics name="evolve_7"  ignore_fitness_equilibrium_check="1" reset_baselines="0" max_accepted_trials="%%n_trials%%"
mover_name="mut" recover_low="0" preapply="0" drift="1" progress_file="trajs/progress_%%id%%_%%e_delta%%_bl_7.txt" filter_name="stability"
trials="100000
00" scorefxn_name="beta_nov16_cart" steepness="0.86" population_size="15848" offset="%%offset%%"/>
  <EvolutionaryDynamics name="evolve_8"  ignore_fitness_equilibrium_check="1" reset_baselines="0" max_accepted_trials="%%n_trials%%"
mover_name="mut" recover_low="0" preapply="0" drift="1" progress_file="trajs/progress_%%id%%_%%e_delta%%_bl_8.txt" filter_name="stability"
trials="100000
00" scorefxn_name="beta_nov16_cart" steepness="0.86" population_size="15848" offset="%%offset%%"/>
  <EvolutionaryDynamics name="evolve_9"  ignore_fitness_equilibrium_check="1" reset_baselines="0" max_accepted_trials="%%n_trials%%"
mover_name="mut" recover_low="0" preapply="0" drift="1" progress_file="trajs/progress_%%id%%_%%e_delta%%_bl_9.txt" filter_name="stability"
trials="100000
00" scorefxn_name="beta_nov16_cart" steepness="0.86" population_size="15848" offset="%%offset%%"/>
  <EvolutionaryDynamics name="evolve_10" ignore_fitness_equilibrium_check="1" reset_baselines="0" max_accepted_trials="%%n_trials%%"
mover_name="mut" recover_low="0" preapply="0" drift="1" progress_file="trajs/progress_%%id%%_%%e_delta%%_bl_10.txt" filter_name="stability"
trials="10000
000" scorefxn_name="beta_nov16_cart" steepness="0.86" population_size="15848" offset="%%offset%%"/>

 </MOVERS>

                                                                                    23,5        Top
<ROSETTASCRIPTS>
    <SCOREFXNS>
        <ScoreFunction name="beta_nov16_cart" weights="beta_nov16_cart"/>
    </SCOREFXNS>

 <FILTERS>
        <ScoreType name="total_score" scorefxn="beta_nov16_cart" score_type="total_score" confidence="0" threshold="100000"/>
        <Sigmoid name="stability" filter="total_score" steepness="0.86" offset="%%offset%%" negate="0"/>
    </FILTERS>

 <MOVERS>
  <NucleotideMutation name="mut" allow_silent="0" bbnbrs="0" fast_relax_cycles="1" flexbb="0" dualspace="1" kappa="2.7" rho="0.1"
scorefxn="beta_nov16_cart"/>
   <NucleotideMutation name="measure_energies0" bbnbrs="0" score_file="ranks/bl_0_%%e_delta%%_%%id%%.txt" allow_silent="1"
fast_relax_cycles="1" measure_e_dist_at_site="9999" flexbb="0" dualspace="1" scorefxn="beta_nov16_cart"/>
  <NucleotideMutation name="measure_energies1" bbnbrs="0" score_file="ranks/bl_1_%%e_delta%%_%%id%%.txt" allow_silent="1"
fast_relax_cycles="1" measure_e_dist_at_site="9999" flexbb="0" dualspace="1" scorefxn="beta_nov16_cart"/>
  <NucleotideMutation name="measure_energies2" bbnbrs="0" score_file="ranks/bl_2_%%e_delta%%_%%id%%.txt" allow_silent="1"
fast_relax_cycles="1" measure_e_dist_at_site="9999" flexbb="0" dualspace="1" scorefxn="beta_nov16_cart"/>
  <NucleotideMutation name="measure_energies3" bbnbrs="0" score_file="ranks/bl_3_%%e_delta%%_%%id%%.txt" allow_silent="1"
fast_relax_cycles="1" measure_e_dist_at_site="9999" flexbb="0" dualspace="1" scorefxn="beta_nov16_cart"/>
  <NucleotideMutation name="measure_energies4" bbnbrs="0" score_file="ranks/bl_4_%%e_delta%%_%%id%%.txt" allow_silent="1"
fast_relax_cycles="1" measure_e_dist_at_site="9999" flexbb="0" dualspace="1" scorefxn="beta_nov16_cart"/>
  <NucleotideMutation name="measure_energies5" bbnbrs="0" score_file="ranks/bl_5_%%e_delta%%_%%id%%.txt" allow_silent="1"
fast_relax_cycles="1" measure_e_dist_at_site="9999" flexbb="0" dualspace="1" scorefxn="beta_nov16_cart"/>
  <NucleotideMutation name="measure_energies6" bbnbrs="0" score_file="ranks/bl_6_%%e_delta%%_%%id%%.txt" allow_silent="1"
fast_relax_cycles="1" measure_e_dist_at_site="9999" flexbb="0" dualspace="1" scorefxn="beta_nov16_cart"/>
  <NucleotideMutation name="measure_energies7" bbnbrs="0" score_file="ranks/bl_7_%%e_delta%%_%%id%%.txt" allow_silent="1"
fast_relax_cycles="1" measure_e_dist_at_site="9999" flexbb="0" dualspace="1" scorefxn="beta_nov16_cart"/>
  <NucleotideMutation name="measure_energies8" bbnbrs="0" score_file="ranks/bl_8_%%e_delta%%_%%id%%.txt" allow_silent="1"
fast_relax_cycles="1" measure_e_dist_at_site="9999" flexbb="0" dualspace="1" scorefxn="beta_nov16_cart"/>
  <NucleotideMutation name="measure_energies9" bbnbrs="0" score_file="ranks/bl_9_%%e_delta%%_%%id%%.txt" allow_silent="1"
fast_relax_cycles="1" measure_e_dist_at_site="9999" flexbb="0" dualspace="1" scorefxn="beta_nov16_cart"/>
  <NucleotideMutation name="measure_energies10" bbnbrs="0" score_file="ranks/bl_10_%%e_delta%%_%%id%%.txt" allow_silent="1"
fast_relax_cycles="1" measure_e_dist_at_site="9999" flexbb="0" dualspace="1" scorefxn="beta_nov16_cart"/>
  NucleotideMutation name="measure_energies_0" bbnbrs="0" score_file="trajs/energies_trial_0_%%id%%.txt" allow_silent="1" fast_relax_cycles="1"
measure_e_dist_at_site="9999" flexbb="0" dualspace="1" scorefxn="beta_nov16_cart"/>
  GenericMonteCarlo name="pre_minimization" reset_baselines="0" mover_name="mut" keep_filters="1" recover_low="1" preapply="0" drift="1"
progress_file="trajs/preminimzation.%%id%%.txt" filter_name="total_score" trials="12000"/>
     <DumpPdb name="dump_1"  fname="dumps/%%id%%_%%e_delta%%_bl_1.pdb" scorefxn="beta_nov16_cart"/>
```

```
      <DumpPdb name="dump_2"  fname="dumps/%%id%%_%%e_delta%%_bl_2.pdb" scorefxn="beta_nov16_cart"/>
      <DumpPdb name="dump_3"  fname="dumps/%%id%%_%%e_delta%%_bl_3.pdb" scorefxn="beta_nov16_cart"/>
      <DumpPdb name="dump_4"  fname="dumps/%%id%%_%%e_delta%%_bl_4.pdb" scorefxn="beta_nov16_cart"/>
      <DumpPdb name="dump_5"  fname="dumps/%%id%%_%%e_delta%%_bl_5.pdb" scorefxn="beta_nov16_cart"/>
      <DumpPdb name="dump_6"  fname="dumps/%%id%%_%%e_delta%%_bl_6.pdb" scorefxn="beta_nov16_cart"/>
      <DumpPdb name="dump_7"  fname="dumps/%%id%%_%%e_delta%%_bl_7.pdb" scorefxn="beta_nov16_cart"/>
      <DumpPdb name="dump_8"  fname="dumps/%%id%%_%%e_delta%%_bl_8.pdb" scorefxn="beta_nov16_cart"/>
      <DumpPdb name="dump_9"  fname="dumps/%%id%%_%%e_delta%%_bl_9.pdb" scorefxn="beta_nov16_cart"/>
      <DumpPdb name="dump_10" fname="dumps/%%id%%_%%e_delta%%_bl_10.pdb" scorefxn="beta_nov16_cart"/>

    <EvolutionaryDynamics name="evolve_0"  ignore_fitness_equilibrium_check="1" reset_baselines="0" max_accepted_trials="%%n_trials%%"
mover_name="mut" recover_low="0" preapply="0" drift="1" progress_file="trajs/progress_%%id%%_%%e_delta%%_bl_0.txt" filter_name="stability"
trials="1000000" scorefxn_name="beta_nov16_cart" steepness="0.86" population_size="15848" offset="%%offset%%"/>
    <EvolutionaryDynamics name="evolve_1"  ignore_fitness_equilibrium_check="1" reset_baselines="0" max_accepted_trials="%%n_trials%%"
mover_name="mut" recover_low="0" preapply="0" drift="1" progress_file="trajs/progress_%%id%%_%%e_delta%%_bl_1.txt" filter_name="stability"
trials="1000000" scorefxn_name="beta_nov16_cart" steepness="0.86" population_size="15848" offset="%%offset%%"/>
    <EvolutionaryDynamics name="evolve_2"  ignore_fitness_equilibrium_check="1" reset_baselines="0" max_accepted_trials="%%n_trials%%"
mover_name="mut" recover_low="0" preapply="0" drift="1" progress_file="trajs/progress_%%id%%_%%e_delta%%_bl_2.txt" filter_name="stability"
trials="1000000" scorefxn_name="beta_nov16_cart" steepness="0.86" population_size="15848" offset="%%offset%%"/>
    <EvolutionaryDynamics name="evolve_3"  ignore_fitness_equilibrium_check="1" reset_baselines="0" max_accepted_trials="%%n_trials%%"
mover_name="mut" recover_low="0" preapply="0" drift="1" progress_file="trajs/progress_%%id%%_%%e_delta%%_bl_3.txt" filter_name="stability"
trials="1000000" scorefxn_name="beta_nov16_cart" steepness="0.86" population_size="15848" offset="%%offset%%"/>
    <EvolutionaryDynamics name="evolve_4"  ignore_fitness_equilibrium_check="1" reset_baselines="0" max_accepted_trials="%%n_trials%%"
mover_name="mut" recover_low="0" preapply="0" drift="1" progress_file="trajs/progress_%%id%%_%%e_delta%%_bl_4.txt" filter_name="stability"
trials="1000000" scorefxn_name="beta_nov16_cart" steepness="0.86" population_size="15848" offset="%%offset%%"/>
    <EvolutionaryDynamics name="evolve_5"  ignore_fitness_equilibrium_check="1" reset_baselines="0" max_accepted_trials="%%n_trials%%"
mover_name="mut" recover_low="0" preapply="0" drift="1" progress_file="trajs/progress_%%id%%_%%e_delta%%_bl_5.txt" filter_name="stability"
trials="1000000" scorefxn_name="beta_nov16_cart" steepness="0.86" population_size="15848" offset="%%offset%%"/>
    <EvolutionaryDynamics name="evolve_6"  ignore_fitness_equilibrium_check="1" reset_baselines="0" max_accepted_trials="%%n_trials%%"
mover_name="mut" recover_low="0" preapply="0" drift="1" progress_file="trajs/progress_%%id%%_%%e_delta%%_bl_6.txt" filter_name="stability"
trials="1000000" scorefxn_name="beta_nov16_cart" steepness="0.86" population_size="15848" offset="%%offset%%"/>
    <EvolutionaryDynamics name="evolve_7"  ignore_fitness_equilibrium_check="1" reset_baselines="0" max_accepted_trials="%%n_trials%%"
mover_name="mut" recover_low="0" preapply="0" drift="1" progress_file="trajs/progress_%%id%%_%%e_delta%%_bl_7.txt" filter_name="stability"
trials="1000000" scorefxn_name="beta_nov16_cart" steepness="0.86" population_size="15848" offset="%%offset%%"/>
    <EvolutionaryDynamics name="evolve_8"  ignore_fitness_equilibrium_check="1" reset_baselines="0" max_accepted_trials="%%n_trials%%"
mover_name="mut" recover_low="0" preapply="0" drift="1" progress_file="trajs/progress_%%id%%_%%e_delta%%_bl_8.txt" filter_name="stability"
trials="1000000" scorefxn_name="beta_nov16_cart" steepness="0.86" population_size="15848" offset="%%offset%%"/>
    <EvolutionaryDynamics name="evolve_9"  ignore_fitness_equilibrium_check="1" reset_baselines="0" max_accepted_trials="%%n_trials%%"
mover_name="mut" recover_low="0" preapply="0" drift="1" progress_file="trajs/progress_%%id%%_%%e_delta%%_bl_9.txt" filter_name="stability"
trials="1000000" scorefxn_name="beta_nov16_cart" steepness="0.86" population_size="15848" offset="%%offset%%"/>
    <EvolutionaryDynamics name="evolve_10" ignore_fitness_equilibrium_check="1" reset_baselines="0" max_accepted_trials="%%n_trials%%"
mover_name="mut" recover_low="0" preapply="0" drift="1" progress_file="trajs/progress_%%id%%_%%e_delta%%_bl_10.txt" filter_name="stability"
trials="1000000" scorefxn_name="beta_nov16_cart" steepness="0.86" population_size="15848" offset="%%offset%%"/>

  </MOVERS>

  <PROTOCOLS>
        <Add mover="measure_energies0"/>
        <Add mover="evolve_1"/>
        <Add mover="measure_energies1"/>
        <Add mover="dump_1"/>
        <Add mover="evolve_2"/>
        <Add mover="measure_energies2"/>
        <Add mover="dump_2"/>
        <Add mover="evolve_3"/>
        <Add mover="measure_energies3"/>
        <Add mover="dump_3"/>
        <Add mover="evolve_4"/>
        <Add mover="measure_energies4"/>
        <Add mover="dump_4"/>
        <Add mover="evolve_5"/>
        <Add mover="measure_energies5"/>
        <Add mover="dump_5"/>
        <Add mover="evolve_6"/>
        <Add mover="measure_energies6"/>
        <Add mover="dump_6"/>
        <Add mover="evolve_7"/>
        <Add mover="measure_energies7"/>
        <Add mover="dump_7"/>
        <Add mover="evolve_8"/>
        <Add mover="measure_energies8"/>
        <Add mover="dump_8"/>
```

```
      <Add mover="evolve_9"/>
      <Add mover="measure_energies9"/>
      <Add mover="dump_9"/>
      <Add mover="evolve_10"/>
      <Add mover="measure_energies10"/>
      <Add mover="dump_10"/>
 </PROTOCOLS>
</ROSETTASCRIPTS>
```

2) Production runs for evolution along a single branch

The structures at the end of the equilibrium trajectories were used as starting point for simulations along a single branch at different offset values. These simulations were run with the following parameters:

kappa=2.7
rho=0.1
The energy function used was "beta_nov16_cart".

The data from these simulations are presented in the section "*The selection pressure impacts the probability distribution over proposed and accepted DDG values*" and **Figure 3** in the main text.

RosettaEvolve production run example:

rosetta_scripts.default.linuxgccrelease -s in/5azu.pdb -parser:protocol equilibrate_sequence.xml @flags -overwrite -out:prefix pdbs/5azu. -parser:script_vars id=5azu e_delta=-427.566 offset=-427.566 n_trials=127

rosetta_scripts.default.linuxgccrelease -s in/5azu.pdb -parser:protocol production_run.xml @flags -overwrite -out:prefix pdbs/5azu. -parser:script_vars id=5azu e_delta=-427.566 offset=-427.566 n_trials=127

with the following XML script, production_run.xml

```
<ROSETTASCRIPTS>
   <SCOREFXNS>
      <ScoreFunction name="beta_nov16_cart" weights="beta_nov16_cart"/>
   </SCOREFXNS>

 <FILTERS>
      <ScoreType name="total_score" scorefxn="beta_nov16_cart" score_type="total_score" confidence="0" threshold="100000"/>
      <Sigmoid name="stability" filter="total_score" steepness="0.86" offset="%%offset%%" negate="0"/>
   </FILTERS>

 <MOVERS>
  <NucleotideMutation name="mut" allow_silent="1" bbnbrs="0" fast_relax_cycles="1" flexbb="0" dualspace="1" kappa="2.7" rho="0.1"
scorefxn="beta_nov16_cart"/>
   <NucleotideMutation name="measure_energies0" bbnbrs="0" score_file="ranks/bl_0_%%e_delta%%_%%id%%.txt" allow_silent="1"
fast_relax_cycles="1" measure_e_dist_at_site="9999" flexbb="0" dualspace="1" scorefxn="beta_nov16_cart"/>
  <NucleotideMutation name="measure_energies1" bbnbrs="0" score_file="ranks/bl_1_%%e_delta%%_%%id%%.txt" allow_silent="1"
fast_relax_cycles="1" measure_e_dist_at_site="9999" flexbb="0" dualspace="1" scorefxn="beta_nov16_cart"/>
  <NucleotideMutation name="measure_energies2" bbnbrs="0" score_file="ranks/bl_2_%%e_delta%%_%%id%%.txt" allow_silent="1"
fast_relax_cycles="1" measure_e_dist_at_site="9999" flexbb="0" dualspace="1" scorefxn="beta_nov16_cart"/>
  <NucleotideMutation name="measure_energies3" bbnbrs="0" score_file="ranks/bl_3_%%e_delta%%_%%id%%.txt" allow_silent="1"
fast_relax_cycles="1" measure_e_dist_at_site="9999" flexbb="0" dualspace="1" scorefxn="beta_nov16_cart"/>
  <NucleotideMutation name="measure_energies4" bbnbrs="0" score_file="ranks/bl_4_%%e_delta%%_%%id%%.txt" allow_silent="1"
fast_relax_cycles="1" measure_e_dist_at_site="9999" flexbb="0" dualspace="1" scorefxn="beta_nov16_cart"/>
  <NucleotideMutation name="measure_energies5" bbnbrs="0" score_file="ranks/bl_5_%%e_delta%%_%%id%%.txt" allow_silent="1"
fast_relax_cycles="1" measure_e_dist_at_site="9999" flexbb="0" dualspace="1" scorefxn="beta_nov16_cart"/>
```

```
  <NucleotideMutation name="measure_energies6" bbnbrs="0" score_file="ranks/bl_6_%%e_delta%%_%%id%%.txt" allow_silent="1"
fast_relax_cycles="1" measure_e_dist_at_site="9999" flexbb="0" dualspace="1" scorefxn="beta_nov16_cart"/>
  <NucleotideMutation name="measure_energies7" bbnbrs="0" score_file="ranks/bl_7_%%e_delta%%_%%id%%.txt" allow_silent="1"
fast_relax_cycles="1" measure_e_dist_at_site="9999" flexbb="0" dualspace="1" scorefxn="beta_nov16_cart"/>
  <NucleotideMutation name="measure_energies8" bbnbrs="0" score_file="ranks/bl_8_%%e_delta%%_%%id%%.txt" allow_silent="1"
fast_relax_cycles="1" measure_e_dist_at_site="9999" flexbb="0" dualspace="1" scorefxn="beta_nov16_cart"/>
  <NucleotideMutation name="measure_energies9" bbnbrs="0" score_file="ranks/bl_9_%%e_delta%%_%%id%%.txt" allow_silent="1"
fast_relax_cycles="1" measure_e_dist_at_site="9999" flexbb="0" dualspace="1" scorefxn="beta_nov16_cart"/>
  <NucleotideMutation name="measure_energies10" bbnbrs="0" score_file="ranks/bl_10_%%e_delta%%_%%id%%.txt" allow_silent="1"
fast_relax_cycles="1" measure_e_dist_at_site="9999" flexbb="0" dualspace="1" scorefxn="beta_nov16_cart"/>
  NucleotideMutation name="measure_energies_0" bbnbrs="0" score_file="trajs/energies_trial_0_%%id%%.txt" allow_silent="1" fast_relax_cycles="1"
measure_e_dist_at_site="9999" flexbb="0" dualspace="1" scorefxn="beta_nov16_cart"/>
  GenericMonteCarlo name="pre_minimization" reset_baselines="0" mover_name="mut" keep_filters="1" recover_low="1" preapply="0" drift="1"
progress_file="trajs/preminimzation.%%id%%.txt" filter_name="total_score" trials="12000"/>
    <DumpPdb name="dump_1"  fname="dumps/%%id%%_%%e_delta%%_bl_1.pdb" scorefxn="beta_nov16_cart"/>
    <DumpPdb name="dump_2"  fname="dumps/%%id%%_%%e_delta%%_bl_2.pdb" scorefxn="beta_nov16_cart"/>
    <DumpPdb name="dump_3"  fname="dumps/%%id%%_%%e_delta%%_bl_3.pdb" scorefxn="beta_nov16_cart"/>
    <DumpPdb name="dump_4"  fname="dumps/%%id%%_%%e_delta%%_bl_4.pdb" scorefxn="beta_nov16_cart"/>
    <DumpPdb name="dump_5"  fname="dumps/%%id%%_%%e_delta%%_bl_5.pdb" scorefxn="beta_nov16_cart"/>
    <DumpPdb name="dump_6"  fname="dumps/%%id%%_%%e_delta%%_bl_6.pdb" scorefxn="beta_nov16_cart"/>
    <DumpPdb name="dump_7"  fname="dumps/%%id%%_%%e_delta%%_bl_7.pdb" scorefxn="beta_nov16_cart"/>
    <DumpPdb name="dump_8"  fname="dumps/%%id%%_%%e_delta%%_bl_8.pdb" scorefxn="beta_nov16_cart"/>
    <DumpPdb name="dump_9"  fname="dumps/%%id%%_%%e_delta%%_bl_9.pdb" scorefxn="beta_nov16_cart"/>
    <DumpPdb name="dump_10" fname="dumps/%%id%%_%%e_delta%%_bl_10.pdb" scorefxn="beta_nov16_cart"/>

  <EvolutionaryDynamics name="evolve_0"  ignore_fitness_equilibrium_check="1" reset_baselines="0" max_accepted_trials="%%n_trials%%"
mover_name="mut" recover_low="0" preapply="0" drift="1" progress_file="trajs/progress_%%id%%_%%e_delta%%_bl_0.txt" filter_name="stability"
trials="1000000" scorefxn_name="beta_nov16_cart" steepness="0.86" population_size="15848" offset="%%offset%%"/>
  <EvolutionaryDynamics name="evolve_1"  ignore_fitness_equilibrium_check="1" reset_baselines="0" max_accepted_trials="%%n_trials%%"
mover_name="mut" recover_low="0" preapply="0" drift="1" progress_file="trajs/progress_%%id%%_%%e_delta%%_bl_1.txt" filter_name="stability"
trials="1000000" scorefxn_name="beta_nov16_cart" steepness="0.86" population_size="15848" offset="%%offset%%"/>
  <EvolutionaryDynamics name="evolve_2"  ignore_fitness_equilibrium_check="1" reset_baselines="0" max_accepted_trials="%%n_trials%%"
mover_name="mut" recover_low="0" preapply="0" drift="1" progress_file="trajs/progress_%%id%%_%%e_delta%%_bl_2.txt" filter_name="stability"
trials="1000000" scorefxn_name="beta_nov16_cart" steepness="0.86" population_size="15848" offset="%%offset%%"/>
  <EvolutionaryDynamics name="evolve_3"  ignore_fitness_equilibrium_check="1" reset_baselines="0" max_accepted_trials="%%n_trials%%"
mover_name="mut" recover_low="0" preapply="0" drift="1" progress_file="trajs/progress_%%id%%_%%e_delta%%_bl_3.txt" filter_name="stability"
trials="1000000" scorefxn_name="beta_nov16_cart" steepness="0.86" population_size="15848" offset="%%offset%%"/>
  <EvolutionaryDynamics name="evolve_4"  ignore_fitness_equilibrium_check="1" reset_baselines="0" max_accepted_trials="%%n_trials%%"
mover_name="mut" recover_low="0" preapply="0" drift="1" progress_file="trajs/progress_%%id%%_%%e_delta%%_bl_4.txt" filter_name="stability"
trials="1000000" scorefxn_name="beta_nov16_cart" steepness="0.86" population_size="15848" offset="%%offset%%"/>
  <EvolutionaryDynamics name="evolve_5"  ignore_fitness_equilibrium_check="1" reset_baselines="0" max_accepted_trials="%%n_trials%%"
mover_name="mut" recover_low="0" preapply="0" drift="1" progress_file="trajs/progress_%%id%%_%%e_delta%%_bl_5.txt" filter_name="stability"
trials="1000000" scorefxn_name="beta_nov16_cart" steepness="0.86" population_size="15848" offset="%%offset%%"/>
  <EvolutionaryDynamics name="evolve_6"  ignore_fitness_equilibrium_check="1" reset_baselines="0" max_accepted_trials="%%n_trials%%"
mover_name="mut" recover_low="0" preapply="0" drift="1" progress_file="trajs/progress_%%id%%_%%e_delta%%_bl_6.txt" filter_name="stability"
trials="1000000" scorefxn_name="beta_nov16_cart" steepness="0.86" population_size="15848" offset="%%offset%%"/>
  <EvolutionaryDynamics name="evolve_7"  ignore_fitness_equilibrium_check="1" reset_baselines="0" max_accepted_trials="%%n_trials%%"
mover_name="mut" recover_low="0" preapply="0" drift="1" progress_file="trajs/progress_%%id%%_%%e_delta%%_bl_7.txt" filter_name="stability"
trials="1000000" scorefxn_name="beta_nov16_cart" steepness="0.86" population_size="15848" offset="%%offset%%"/>
  <EvolutionaryDynamics name="evolve_8"  ignore_fitness_equilibrium_check="1" reset_baselines="0" max_accepted_trials="%%n_trials%%"
mover_name="mut" recover_low="0" preapply="0" drift="1" progress_file="trajs/progress_%%id%%_%%e_delta%%_bl_8.txt" filter_name="stability"
trials="1000000" scorefxn_name="beta_nov16_cart" steepness="0.86" population_size="15848" offset="%%offset%%"/>
  <EvolutionaryDynamics name="evolve_9"  ignore_fitness_equilibrium_check="1" reset_baselines="0" max_accepted_trials="%%n_trials%%"
mover_name="mut" recover_low="0" preapply="0" drift="1" progress_file="trajs/progress_%%id%%_%%e_delta%%_bl_9.txt" filter_name="stability"
trials="1000000" scorefxn_name="beta_nov16_cart" steepness="0.86" population_size="15848" offset="%%offset%%"/>
  <EvolutionaryDynamics name="evolve_10" ignore_fitness_equilibrium_check="1" reset_baselines="0" max_accepted_trials="%%n_trials%%"
mover_name="mut" recover_low="0" preapply="0" drift="1" progress_file="trajs/progress_%%id%%_%%e_delta%%_bl_10.txt" filter_name="stability"
trials="1000000" scorefxn_name="beta_nov16_cart" steepness="0.86" population_size="15848" offset="%%offset%%"/>

 </MOVERS>

 <PROTOCOLS>
      Add mover="measure_energies0"/>
      <Add mover="evolve_1"/>
      Add mover="measure_energies1"/>
      <Add mover="dump_1"/>
      <Add mover="evolve_2"/>
      Add mover="measure_energies2"/>
      <Add mover="dump_2"/>
      <Add mover="evolve_3"/>
      Add mover="measure_energies3"/>
      <Add mover="dump_3"/>
```

```
        <Add mover="evolve_4"/>
        Add mover="measure_energies4"/>
        <Add mover="dump_4"/>
        <Add mover="evolve_5"/>
        Add mover="measure_energies5"/>
        <Add mover="dump_5"/>
        <Add mover="evolve_6"/>
        Add mover="measure_energies6"/>
        <Add mover="dump_6"/>
        <Add mover="evolve_7"/>
        Add mover="measure_energies7"/>
        <Add mover="dump_7"/>
        <Add mover="evolve_8"/>
        Add mover="measure_energies8"/>
        <Add mover="dump_8"/>
        <Add mover="evolve_9"/>
        Add mover="measure_energies9"/>
        <Add mover="dump_9"/>
        <Add mover="evolve_10"/>
        Add mover="measure_energies10"/>
        <Add mover="dump_10"/>
 </PROTOCOLS>
</ROSETTASCRIPTS>
```

3) Production runs for evolution along a single branch with a single mutation

Simulations are carried out with a corresponding xml scripts as for the production run, but with a single mutation per trajectory.

The result of these simulations is presented in the section "*Fluctuations in protein stability result in fluctuations in site rates*" in the main manuscript and **Figure 7.**

Example command line:

rosetta_scripts.default.linuxgccrelease -s in/5azu.pdb -parser:protocol single_mutation_only.xml @flags -overwrite -out:prefix pdbs/5azu. -parser:script_vars id=5azu e_delta=-427.566 offset=-427.566 n_trials=1

4) Evolution of a protein sequence along a phylogenetic tree

Evolutionary trajectories were run using a python wrapper script that recursively visits all nodes in the tree and calls RosettaEvolve. The wrapper script takes as input an offset and a mutation rate (determined in the production run).

Python rosetta_evolution_wrapper_mpi.py --offset -342.566 --mutationRate 9.38897e-05

The script takes the desired offset for the simulation and the estimated amino acid mutation rate. The mutation rate is estimated from the production run and is used together with the branch length in the phylogenetic tree to decide on the number mutational trials.

The result of the tree crawling simulation is presented in the section "*A strong covariation signal is found when phylogenetic trees are simulated by RosettaEvolve*" and **Figure 4,5 and 6** in the main manuscript.

The rosetta_evolution_wrapper_mpi.py script calls Rosetta inside the using a XML script, simulate_branch_expected.xml.

```
<ROSETTASCRIPTS>
    <SCOREFXNS>
        <ScoreFunction name="beta_nov16_cart" weights="beta_nov16_cart"/>
    </SCOREFXNS>

    <TASKOPERATIONS>
        <InitializeFromCommandline name="init"/>
        <RestrictToRepacking name="rtr"/>
    </TASKOPERATIONS>

    <FILTERS>
        <ScoreType name="total_score" scorefxn="beta_nov16_cart" score_type="total_score" confidence="0" threshold="100000"/>
        <Sigmoid name="stability" filter="total_score" steepness="0.86" offset="%%offset%%" negate="0"/>
        <Operator name="objective_function" filters="stability" operation="PRODUCT" negate="1" logarithm="1" threshold="100000"/>
    </FILTERS>

    <MOVERS>
        NucleotideMutation name="mut" allow_silent="1" flexbb="1" bbnbrs="999" scorefxn="beta_nov16_cart"/> I need cont_if_silent=0 here, as this
will be used for rate matrix calculations.
        <NucleotideMutation name="mut" allow_silent="1" bbnbrs="0" fast_relax_cycles="1" flexbb="0" dualspace="1" kappa="2.7" rho="0.1"
scorefxn="beta_nov16_cart"/>
        <EvolutionaryDynamics name="evolve" ignore_fitness_equilibrium_check="1" reset_baselines="0" mover_name="mut" recover_low="0"
preapply="0" drift="1" progress_file="%%progress_id%%.txt" filter_name="stability" branch_length="%%branch_length%%"
mutation_rate="%%mutationRate%%" scorefxn_name="beta_nov16_cart" steepness="0.86" population_size="15848" offset="%%offset%%"/>
   </MOVERS>

    <PROTOCOLS>
    <Add mover="evolve"/>
    </PROTOCOLS>
</ROSETTASCRIPTS>
```
